# Ikaros cooperates with Notch activation and antagonizes TGFβ signaling to promote pDC development

**DOI:** 10.1371/journal.pgen.1007485

**Published:** 2018-07-12

**Authors:** Jérôme Mastio, Célestine Simand, Giovanni Cova, Philippe Kastner, Susan Chan, Peggy Kirstetter

**Affiliations:** 1 Institut de Génétique et de Biologie Moléculaire et Cellulaire (IGBMC), INSERM U1258, CNRS UMR 7104, Université de Strasbourg, Illkirch, France; 2 Faculté de Médecine, Université de Strasbourg, Strasbourg, France; Centre for Cancer Biology, SA Pathology, AUSTRALIA

## Abstract

Plasmacytoid and conventional dendritic cells (pDCs and cDCs) arise from monocyte and dendritic progenitors (MDPs) and common dendritic progenitors (CDPs) through gene expression changes that remain partially understood. Here we show that the Ikaros transcription factor is required for DC development at multiple stages. Ikaros cooperates with Notch pathway activation to maintain the homeostasis of MDPs and CDPs. Ikaros then antagonizes TGFβ function to promote pDC differentiation from CDPs. Strikingly, Ikaros-deficient CDPs and pDCs express a cDC-like transcriptional signature that is correlated with TGFβ activation, suggesting that Ikaros is an upstream negative regulator of the TGFβ pathway and a repressor of cDC-lineage genes in pDCs. Almost all of these phenotypes can be rescued by short-term in vitro treatment with γ-secretase inhibitors, which affects both TGFβ-dependent and -independent pathways, but is Notch-independent. We conclude that Ikaros is a crucial differentiation factor in early dendritic progenitors that is required for pDC identity.

## Author summary

Dendritic cells (DCs) are an important component of the immune system, and exist as two major subtypes: conventional DCs (cDCs) which present antigen via major histocompatibility class II molecules, and plasmacytoid DCs (pDCs) which act mainly as producers of type-I interferon in response to viral infections. Both types of DCs derive from a common dendritic progenitor (CDP), but the genetic pathways that influence their development are not completely understood. A better understanding of these pathways is important, which may lead to protocols for generating specific DCs in culture, depending on the need. In this study, we have discovered important roles for the Ikaros transcription factor in DC development. We found that: (i) Ikaros cooperates with the Notch pathway to promote the development or homeostasis of CDPs; (ii) Ikaros controls pDC differentiation from CDPs through a γ-secretase sensitive pathway; and (iii) Ikaros antagonizes the TGFβ pathway to inhibit cDC differentiation. Our results thus identify Ikaros as a key player in the early steps of DC development.

## Introduction

Dendritic cells (DCs) are essential modulators of the immune response [1]. They can be broadly divided into conventional DCs (cDC), which are required for antigen presentation, and plasmacytoid DCs (pDC), which secrete high quantities of type-I interferon (IFN-α, -β, -ω) upon certain viral infections [2, 3]. cDCs are further divided into cDC1 (CD8^+^) and cDC2 (CD11b^+^) subsets. Both DC lineages develop in the bone marrow. Monocyte and dendritic progenitors (MDPs) are the earliest known DC precursors, and they give rise to monocytes and common dendritic progenitors (CDPs) [4–6]. In turn, CDPs differentiate into pDCs and pre-cDCs, the latter of which migrate to the periphery to become cDCs. The molecular circuits regulating DC cell fate have been intensively studied, and some transcriptional regulators (Ikaros, E2.2, PU.1, IRF8, GFI1, NFIL3, BATF3, BCL11a) and canonical signaling pathways (TGFβ, Notch, Wnt) have been identified [3, 7–12]. However, the relationships and interactions between these players remain unclear, and this is important to understand if we wish to manipulate DC function.

Deficiency of the Ikaros zinc finger DNA-binding protein and tumor suppressor, encoded by the *Ikzf1* gene, is associated with profoundly impaired DC development. Mice homozygous for a dominant-negative (dn) *Ikzf1* mutation lack all cDCs, while animals with a null mutation predominantly lack cDC2s [13]. In contrast, mice carrying the hypomorphic Ik^L/L^ mutation show a selective block in bone marrow (BM) pDC development, leading to an absence of peripheral pDCs, although cDCs appear normal [14]. These studies highlight the sensitivity of the DC lineages to Ikaros levels, where pDC development requires more Ikaros function than cDCs. In man, patients with germline *IKZF1* mutations also exhibit reduced pDC, but not cDC numbers, indicating a conserved role for Ikaros in DC development [15]. Interestingly, *IKZF1* deletions are associated with blastic plasmacytoid dendritic cell neoplasms (BPDCN), a malignancy of pDC precursors with poor prognosis [16–18]. Thus Ikaros is required for DC development, but little is known about its molecular mechanisms.

Here we show that Ikaros deficiency leads to multiple defects in pDC and cDC development. In particular, Ikaros is required for CDP development, where it antagonizes TGFβ function to promote pDC differentiation. We further show that Ikaros cooperates with Notch pathway activation to support the homeostasis of DC progenitors. Lastly, we show that a transient incubation of bone marrow cells with γ-secretase inhibitors rescues pDC development from WT and Ikaros-deficient BM progenitors, revealing a potentially novel way to enhance pDC function.

## Results

### Ikaros is required for CDP differentiation

To determine how pDC differentiation is affected by Ikaros deficiency, we evaluated DC progenitor populations in Ik^L/L^ mice. Ik^L/L^ cells express functional Ikaros proteins at ~10% of WT levels, and although Ik^L/L^ mice die from Notch-dependent T cell leukemias at 4–6 months of age, the animals studied (6–8 weeks of age) showed no signs of transformation (normal CD4/CD8 profiles, T cell receptor chain usage, Notch pathway activation) [19–21].

**Fig 1:**
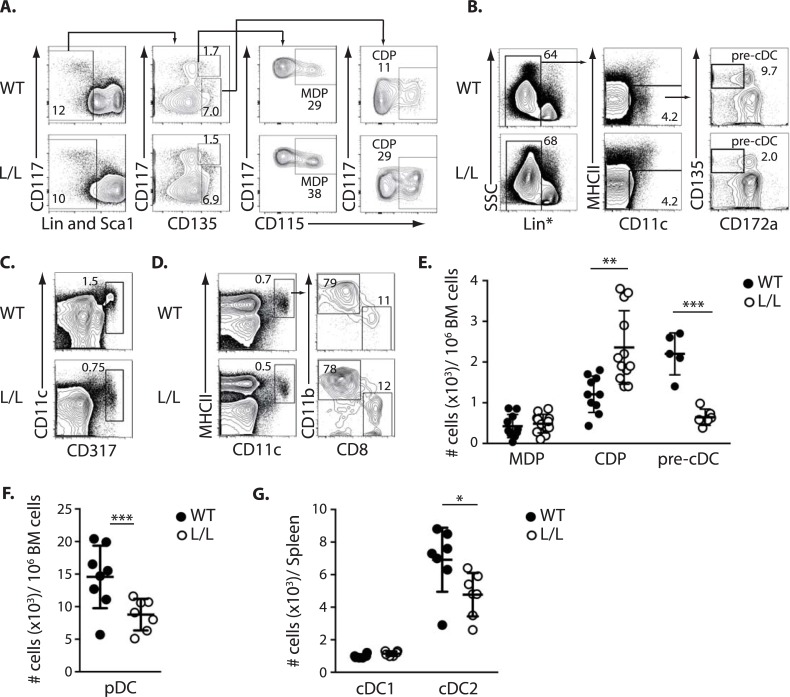
Ikaros regulates DC progenitor development. **(A)** Representative analysis of MDPs, CDPs, **(B)** pre-cDCs, and **(C)** pDCs from Ik^L/L^ (L/L) and WT BM, by flow cytometry. **(D)** Representative analysis of splenic cDCs (CD11c^+^MHCII^+^). **(E)** Relative numbers of BM MDPs, CDPs and pre-cDCs (as gated in A and B), **(F)** BM pDCs (as gated in C), and **(G)** splenic cDCs (as gated in D). Mean±SD of 4–12 animals per group. *p≤0.05; **p≤0.01; ***p≤0.001 (Student’s t-test).

Successive stages of DC development were analyzed, which included BM Lin^-^Sca1^-^CD135^+^ cells, containing CD117^hi^CD115^+^ MDPs and CD117^lo^CD115^+^ CDPs, as well as the more downstream BM CD11c^+^CD317^+^ pDCs and CD11c^+^CD135^+^MHCII^-^CD172a^-^ pre-cDCs, and splenic cDCs (Fig 1) [4–6, 22]. In the BM, CDP numbers were significantly increased and pDC and pre-cDC numbers were significantly decreased in Ik^L/L^ mice, suggesting that Ik^L/L^ DC differentiation is blocked at the CDP stage (Fig 1A–1C, 1E and 1F). In the spleen, Ik^L/L^ animals had no detectable pDCs, as previously reported [13, 14], fewer cDC2s, but similar numbers of cDC1s compared with WT (Fig 1D and 1G). Thus Ikaros deficiency results in the specific accumulation of BM CDPs.

### The Notch pathway is activated in Ikaros-deficient pDCs

**Fig 2:**
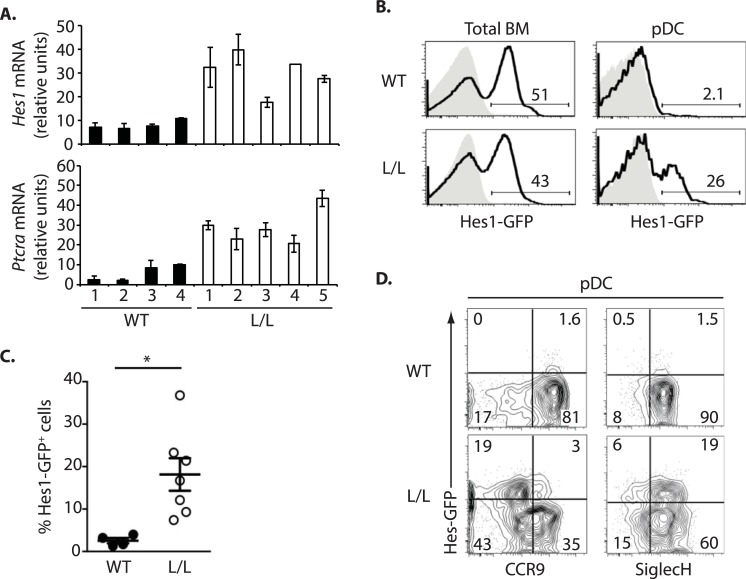
Notch pathway activation in Ik^L/L^ pDCs. **(A)** *Hes1* and *Ptcra* mRNA expression in BM pDCs from 4 WT and 5 (Ik^L/L^) mice, as analyzed by RT-qPCR and normalized to *Hprt* mRNA levels (mean±SD of triplicate data). (**B)** GFP reporter expression (black line) in total BM cells and BM pDCs (CD11c^+^CD317^+^) from Hes1-GFP^+^ WT and Ik^L/L^ mice, by flow cytometry. Grey histograms correspond to control cells from mice lacking the Hes1-GFP reporter. **(C)** Percentage of GFP^+^ BM pDCs (CD11c^+^CD317^+^) from Hes1-GFP^+^ WT and Ik^L/L^ mice, as analyzed in (B). *p≤0.05 (Student’s t-test). **(D)** CCR9 and SiglecH vs. GFP expression in BM pDCs from Hes1-GFP^+^ WT and Ik^L/L^ mice. Representative of 3 independent experiments.

We previously observed in a genome-wide study that genes associated with the Notch pathway (eg. *Hes1*, *Ptcra*, *Uaca*) are upregulated in the BM pDCs of Ik^L/L^ mice [14]. Higher Hes1 and Ptcra mRNA levels were confirmed by RT-qPCR (Fig 2A). To determine if Ikaros deficiency results in Notch activation throughout pDC development, we crossed Ik^L/L^ mice with animals carrying a *Hes1-GFP* knock-in (KI) reporter [23]. Total BM cells from Ik^+/+^ (WT) and Ik^L/L^ Hes1-GFP KI mice contained similar frequencies of GFP^+^ cells (mostly CD19^+^ B cells) (Fig 2B and 2C). In contrast, GFP^+^ cells were nearly absent in WT BM pDCs, but they were present in a fraction of Ik^L/L^ pDCs (7–35%). Ik^L/L^ GFP^+^ pDCs were mostly SiglecH^+^CCR9^lo^, suggesting an immature phenotype (Fig 2D) [24, 25]. CCR9^lo^ pDCs from WT Hes1-GFP KI mice did not express GFP. These data indicated that the *Hes1* locus, and perhaps the Notch pathway, are ectopically activated during pDC development in the mutant mice.

### γ-secretase inhibitors rescue Ik^L/L^ CDP differentiation in vitro and in vivo

**Fig 3:**
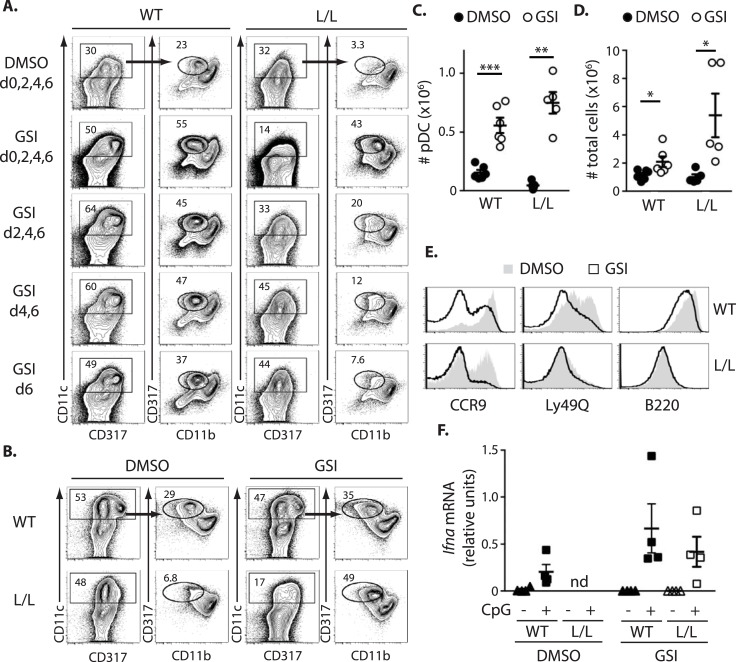
Inhibition of γ-secretase rescues Ik^L/L^ pDC differentiation in vitro. **(A)** Percentage of CD11c^+^CD317^+^CD11b^-^ pDCs after 8 days of Flt3L-supplemented WT and Ik^L/L^ BM cultures, treated with GSI or vehicle (DMSO) at the indicated days of culture. Representative of >5 independent experiments. **(B)** Percentage of pDCs from Flt3L-supplemented WT and Ik^L/L^ BM cultures, treated with GSI or DMSO at day 0 and analyzed at day 8. **(C, D)** Numbers of pDCs (C) and total cell numbers (D) obtained from cultures described in (B). *p≤0.05; **p≤0.01; ***p≤0.001 (Student’s t-test). **(E)** CCR9, Ly49Q and B220 expression on pDCs cultured as in (B). Representative of 3 independent experiments. **(F)** RT-qPCR analysis of *Ifna* expression induced from pDCs after in vitro culture. WT and Ik^L/L^ pDCs were sorted at d8 of culture, after GSI treatment at day 0, and stimulated for 16h with CpG ODN 1585. *Ifna* mRNA levels were measured by RT-qPCR and normalized to *Ubb* mRNA. nd: not done.

To determine if ectopic Notch activation interferes with pDC differentiation in Ik^L/L^ mice, we first blocked Notch signaling in Flt3L-supplemented cultures of total BM cells, using a γ-secretase inhibitor (GSI, Compound E) [26, 27]. As γ-secretase is required to cleave and activate ligand-bound Notch receptors, GSIs are potent inhibitors of Notch function. In the absence of GSI (DMSO), WT cultures generated robust numbers of CD11c^+^CD137^+^CD11b^-^ pDCs over an 8-day period, while Ik^L/L^ cultures did not (Fig 3A). Strikingly, GSI treatment significantly enhanced WT pDC differentiation, and rescued pDC development in the Ik^L/L^ cultures to levels of WT cells. This occurred early, as GSI treatment at day 0 was both necessary and sufficient to rescue Ik^L/L^ pDC development (Fig 3B and 3C). Similar results were obtained with other GSI compounds (DAPT and MRK003). In addition, early GSI treatment resulted in an increase in total cell numbers (Fig 3D), which correlated with an expansion of immature CD11c^-^ cells, particularly in the Ik^L/L^ cultures (Fig 3B). The pDCs produced in the GSI-treated cultures were more immature, and expressed low levels of CCR9 and Ly49Q (Fig 3E); B220 levels, however, remained unchanged after GSI treatment. Importantly, the GSI-rescued WT and Ik^L/L^ pDCs expressed mRNA for *Ifna* following TLR9 stimulation in vitro with CpG ODN 1885 (Fig 3F), suggesting functionality. Because GSI treatment at day 0 of culture was sufficient to induce differentiation, GSI was added only once at the onset of culture in subsequent experiments.

**Fig 4:**
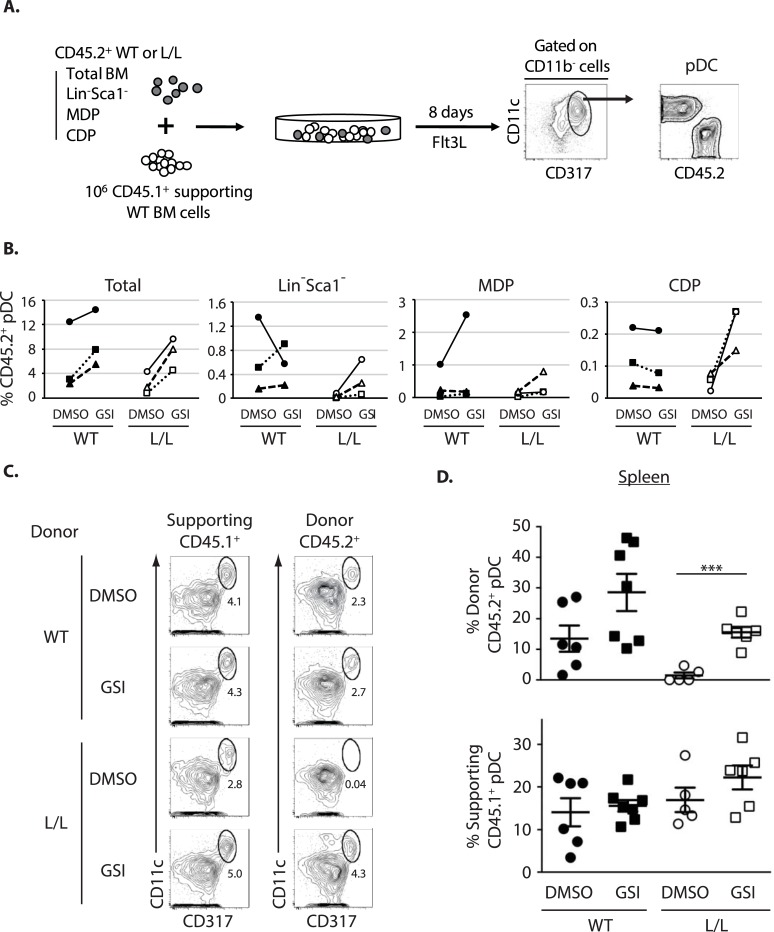
GSI acts on DC progenitors and rescues Ik^L/L^ pDC maturation in vivo. **(A)** Experimental scheme: the indicated cell populations from WT or Ik^L/L^ BM (CD45.2^+^) were cultured with supporting C57BL/6^CD45.1^ (CD45.1^+^) WT BM cells. Cultures were treated with GSI or DMSO at d0, and the percentage of CD45.2^+^ pDCs were analyzed at d8. **(B)** Percentage of CD45.2^+^ pDCs (CD11c^+^CD317^+^CD11b^-^) after 8 days of co-culture. Data from cells of the same mouse treated with DMSO or GSI were connected by lines. Data from 3 independent experiments. **(C)** Representative analysis of splenic pDCs from CD45.2^+^ WT or Ik^L/L^ BM cells, cultured with Flt3L in the presence or absence of GSI for 2d, and then transplanted (2x10^5^ cells per recipient) into lethally-irradiated CD45.1^+^CD45.2^+^ recipient mice, in the presence of supporting CD45.1^+^ WT BM cells (2x10^5^ cells). The presence of pDCs was analyzed 9 days after transplantation. Representative of 2 independent experiments, 2–4 animals per condition per experiment. **(D)** Frequency of splenic CD45.1^+^ and CD45.2^+^ pDCs (CD11c^+^CD317^+^CD11b^-^) in the recipient mice, as described in (A). ***p≤0.001 (Student’s t-test).

To identify the DC progenitor cells sensitive to GSI, we co-cultured WT and Ik^L/L^ total BM cells, purified Lin^-^Sca1^-^ cells, MDPs or CDPs (all CD45.2^+^), with CD45.1^+^ supporting WT BM cells, in the presence of GSI and Flt3L, for 8 days (Fig 4A). The ability of the different CD45.2^+^ populations to give rise to pDCs was evaluated. GSI treatment consistently increased pDC differentiation from Ik^L/L^ CDPs (Fig 4B). On the contrary, GSI did not affect WT MDPs (2 out of 3 experiments) and CDPs, even though it enhanced pDC development from total WT BM cells. We also analyzed Lin^-^Sca1^-^CD117^lo^CD135^+^CD115^-^ cells ("CD115^-^ CDPs") in these assays, as they were reported to contain pDC-specific precursors [28], even though they existed in similar numbers in WT and Ik^L/L^ BM (S1A and S1B Fig); GSI did not affect the pDC production from either WT or Ik^L/L^ CD115^-^ CDPs (S1C and S1D Fig), and these cells were not studied further. These results therefore suggested that Ikaros negatively regulates a γ-secretase-sensitive pathway mainly in (CD115^+^) CDPs.

To determine if transient GSI treatment rescues Ik^L/L^ pDC development in vivo, we adoptively transferred GSI-treated BM cells into recipient mice. WT and Ik^L/L^ BM cells (CD45.2^+^) were cultured with Flt3L and GSI for 2 days, and then transplanted into irradiated hosts (CD45.1^+^CD45.2^+^) along with CD45.1^+^ supporting WT BM cells. BM and spleen cells were analyzed 9 days later for CD45.2^+^ pDCs (Fig 4C and 4D). In the BM, Ik^L/L^ cells generated few CD11c^+^CD137^+^CD11b^-^ pDCs, regardless of GSI treatment (S2 Fig). However, in the spleen, GSI-treated Ik^L/L^ cells generated CD11c^+^CD137^+^CD11b^-^ pDCs while the DMSO-treated cells did not (Fig 4C and 4D). WT cells generated slightly more pDCs after GSI treatment compared with DMSO. Importantly, the CD45.1^+^ supporting cells produced similar frequencies of pDCs in all conditions, indicating that GSI treatment enhanced Ik^L/L^ and WT pDC differentiation in a cell-intrinsic manner.

Collectively, our results indicated that γ-secretase inhibitors rescue Ikaros-deficient pDC development in vitro and in vivo.

### GSI promotes CDP differentiation via Notch-independent pathways

**Fig 5:**
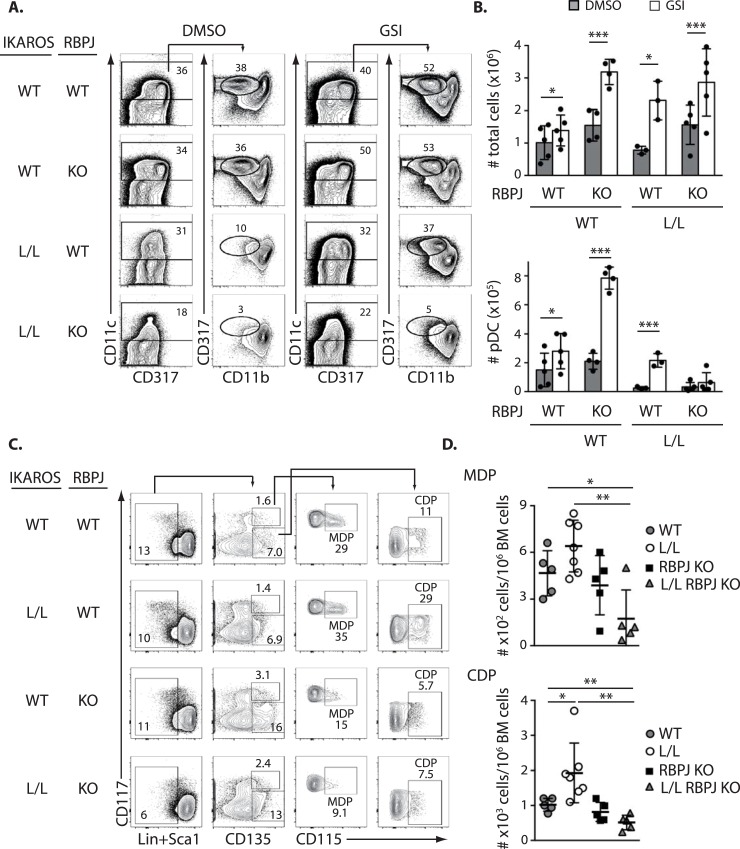
Genetic deletion of RBPJ does not rescue Ik^L/L^ pDC differentiation. **(A)** Representative analysis of Flt3L-supplemented cultures of BM cells from compound mutant mice with Ik^L/L^ and/or RBPJ KO alleles, after addition of DMSO or GSI at day 0. Cultures were analyzed 8d later. **(B)** Numbers of total cells and pDCs obtained from cultures described in (A) (mean±SD from 3–5 mice per genotype; p values were obtained by paired Student’s t-test). **(C)** Analysis and **(D)** relative numbers of MDPs and CDPs from the BM of Ikaros-RBPJ compound mutant mice (representative of 2 independent experiments with 2–5 mice per genotype and per experiment; p values were obtained by Student’s t-test). *p≤0.05; **p≤0.01; ***p≤0.001.

Because γ-secretase inhibitors affect other pathways in addition to Notch, we tested the role of Notch activation in pDC development by genetic means. Ik^L/L^ mice were crossed with animals carrying a floxed null allele for *Rbpj* (Rbpj^f/f^), the Notch transcriptional mediator, and the R26-CreERT2 transgene [29, 30]. Ik^L/L^ Rbpj^+/+^ Cre^+^ (Ik^L/L^ RBPJ WT) and Ik^L/L^ Rbpj^f/f^ Cre^+^ (Ik^L/L^ RBPJ KO) mice, along with control animals, were treated with tamoxifen for 5 days to delete *Rbpj*, and analyzed 5 days after the last injection. Deletion was confirmed by Western blot (S3 Fig). BM cells from the tamoxifen-treated mice were cultured with Flt3L for 8 days, in the presence or absence of GSI, and cell expansion and pDC development were studied (Fig 5A and 5B). In these experiments, we reasoned that if GSI rescues pDC development by inhibiting Notch signaling, then (i) *Rbpj* inactivation should mimic the effects of GSI, and (ii) GSI should not have additional effects when *Rbpj* is deleted.

When cell numbers were evaluated, we observed that the samples treated with GSI contained significantly higher numbers of cells, regardless of RBPJ and/or Ikaros status (Fig 5B). This suggested that GSI inhibits the function of pathways other than Notch. Likewise, when pDC development was evaluated (Fig 5A and 5B), *Rbpj* deletion by itself did not enhance the differentiation of Ik^WT^ DMSO-treated cells, indicating that Notch activation is not required to limit pDC development when Ikaros is present. Further, in Ik^WT^ cells, GSI treatment enhanced pDC differentiation in both RBPJ WT and KO conditions, suggesting that GSI enhances pDC differentiation in the absence of Notch. Interestingly, when similar experiments were performed in Ik^L/L^ conditions, GSI treatment rescued pDC development in the RBPJ WT samples, as expected, but no rescue was observed when both RBPJ and Ikaros were mutated. GSI nevertheless still increased total cell numbers in the cultures from the RBPJ-Ikaros double mutant cells, indicating that its effects on pDC differentiation and cell expansion are separable.

To determine why GSI treatment did not rescue pDC development in Ik^L/L^ RBPJ KO BM cultures, we analyzed the BM DC progenitor populations of tamoxifen-treated Ik^L/L^ RBPJ KO mice and littermate controls (Fig 5C and 5D). Specifically, we evaluated the CDP population in the double mutant mice, as GSI rescues Ik^L/L^ CDP differentiation. These experiments revealed that MDPs and CDPs were barely detectable in most of the Ik^L/L^ RBPJ KO BM samples (4 out of 5), while the BM from single mutant mice contained easily recognizable MDP and CDP cells. These results indicated that the GSI target population is absent in the Ik^L/L^ RBPJ KO BM, and suggested that Ikaros and Notch activation cooperate to generate or maintain MDP and CDP cells in the BM.

Collectively, our results demonstrate that GSI treatment inhibits a Notch-independent pathway important for CDP development.

### The TGFβ pathway is activated in Ikaros-deficient CDPs

To further investigate the molecular pathways targeted by Ikaros and γ-secretase in CDPs, we studied the transcriptome profiles of WT and Ik^L/L^ MDPs and CDPs, cultured in the presence or absence of GSI. We used a protocol similar to the one above, and co-cultured CD45.2^+^ WT or Ik^L/L^ MDPs, and CDPs, with supporting CD45.1^+^ WT BM cells. CD45.2^+^ cells were purified after 24h, and their transcriptomes were analyzed by microarray.

**Fig 6:**
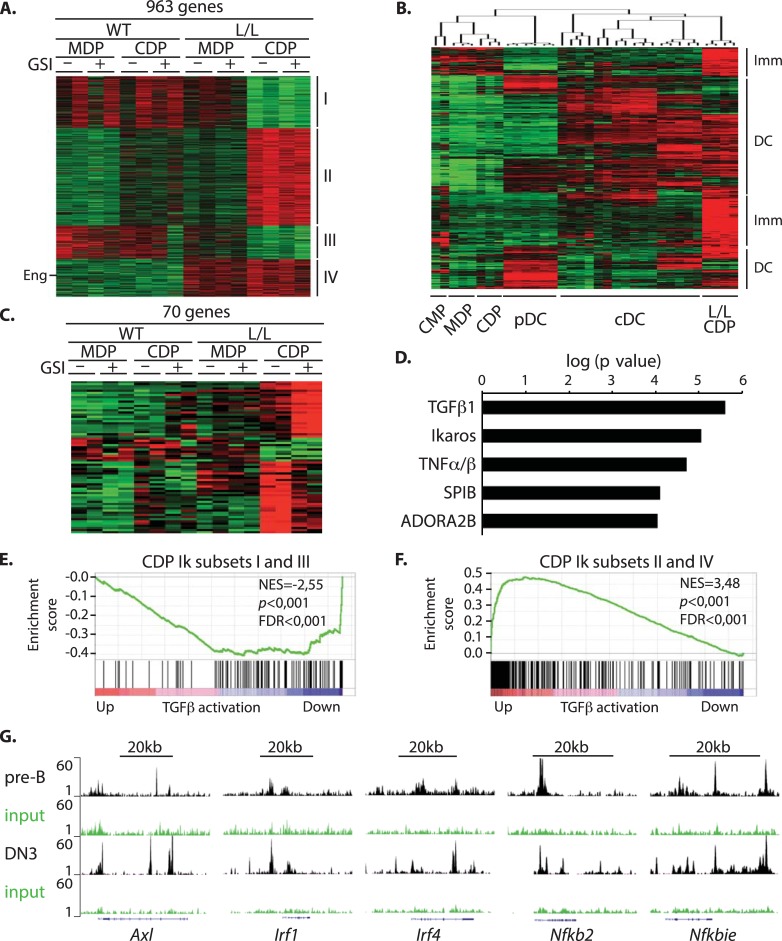
The TGFβ1 pathway is activated in Ik^L/L^ CDPs. CD45.2^+^ WT or Ik^L/L^ MDPs, and CDPs, were co-cultured with supporting CD45.1^+^ WT BM cells for 24h with Flt3L and GSI (or DMSO). CD45.2^+^ cells were then re-purified and their transcriptomes analyzed. 2 mice per condition. **(A)** Heat map representing K-means clustering of 963 genes differentially expressed between WT or Ik^L/L^ CDPs [fold change (FC)>1.5]. Clusters I and II are deregulated specifically in Ik^L/L^ CDPs. Clusters III and IV are deregulated in all Ik^L/L^ DC progenitors. Eng indicates the Endoglin gene. Red and green indicate high and low expression, respectively. **(B)** Hierarchical clustering of the genes from clusters II and IV in (A), using Immgen transcriptome data for DC progenitors and mature subsets (GSE15907). Clusters of genes similarly expressed between Ik^L/L^ CDPs and DC progenitors (Imm) or mature DCs (DC) are indicated. **(C)** K-means clustering of 70 genes differentially expressed between WT and Ik^L/L^ CDPs, and deregulated by GSI (FC>1.5). **(D)** Top 5 putative upstream regulators related to the 70 genes from (C), as identified by the Ingenuity Pathways Analysis software. **(E)** GSEA enrichment plots of genes specifically down-regulated [clusters I and III in (A)] and **(F)** up-regulated (clusters II and IV) in Ik^L/L^ CDPs. The ranked gene list corresponds to TGFβ1-regulated genes in CDPs, as identified by Felker et al (2010). NES: normalized enrichment score; FDR: false discovery rate. **(G)** Genome browser tracks showing Ikaros binding to loci associated with TGFβ activation in pre-B cells (BH1-Ik1-ER-Bcl2 cell line) and immature DN3 thymocytes (GEO GSE114629 and GSE61148 accession numbers).

In the vehicle-treated samples, 963 genes were differentially expressed >1.5-fold between Ik^L/L^ CDPs and all WT populations (Fig 6A). Approximately 30% of these genes were deregulated in both Ik^L/L^ MDPs and CDPs (clusters III and IV), and 70% were deregulated only in the Ik^L/L^ CDPs (clusters I and II). To determine how these genes are expressed during WT DC development, we compared their levels of expression in Ik^L/L^ CDPs with those in WT progenitors and mature DC populations, as reported by the ImmGen Compendium (GSE15907), using unsupervised clustering [7]. Interestingly, this revealed that, among the genes up-regulated in Ik^L/L^ CDPs (Fig 6B), the large majority (>70%) were related to mature cDC genes, and not pDCs. The remainder of the genes were DC progenitor-related. In contrast, among the genes down-regulated in Ik^L/L^ CDPs (S4A Fig), most were related to DC progenitor (CMP, MDP, CDP) genes. Further, gene set enrichment analyses (GSEA) indicated that both the up- and down-regulated genes in the Ik^L/L^ CDPs correlated with those normally expressed in WT cDCs (S4B Fig). Thus, Ikaros is required to repress the premature expression of cDC-associated genes in CDPs. We then asked if the cDC transcriptional hallmarks that characterize the Ik^L/L^ CDPs were also retained in the BM pDCs from Ik^L/L^ mice. Indeed, GSEA analysis showed that genes up- or down-regulated in Ik^L/L^ pDCs (transcriptome data from [14]) were also up- or down-regulated in mature cDCs (S4C Fig), thereby confirming our hypothesis.

Among the genes deregulated in Ik^L/L^ CDPs, only 70 were differentially expressed between GSI and DMSO treated samples (Fig 6C, S1 Table). To identify the potential upstream pathways involved in the regulation of their expression in DC progenitors, we performed Ingenuity Pathway Analysis (Fig 6D). This revealed Ikaros to be a significant probable regulator, which validated our approach, and showed the importance of Ikaros in CDPs. The top candidate, however, was TGFβ1, which was interesting because TGFβ1 was previously reported to skew CDP differentiation towards the cDC lineage at the expense of pDCs [9]. We therefore asked if the deregulated genes found in Ik^L/L^ CDPs were enriched for TGFβ-associated genes, by GSEA. These results showed a strong and direct correlation between the genes down-regulated in Ik^L/L^ CDPs and those down-regulated by TGFβ1 signaling (Fig 6E) [9]. Conversely, the genes up-regulated in Ik^L/L^ CDPs were up-regulated by TGFβ1 activation (Fig 6F). Thus, Ikaros expression is correlated with reduced TGFβ1 signaling in CDP cells.

To determine if Ikaros directly regulates the TGFβ pathway, we investigated its capacity to bind TGFβ target genes. The low number of CDPs in WT mice did not allow us to directly investigate Ikaros binding in these cells. We therefore compared Ikaros binding to chromatin from 2 unrelated precursor cell types (pre-B cells and DN3 thymocytes) [31, 32], because conserved binding might indicate that Ikaros regulates similar elements across hematopoietic cell types. These analyses showed strong and conserved Ikaros binding to several TGFβ target genes implicated in DC differentiation (eg. *Axl*, *Irf1*, *Irf4*, *Nfkb2*, *Nfkbie*, *Rel*, *Relb*) (Fig 6G and S4 Fig), and suggested that Ikaros may directly regulate the expression of TGFβ target genes in CDPs.

### Inhibition of TGFβ signaling rescues Ikaros-deficient pDC development

**Fig 7:**
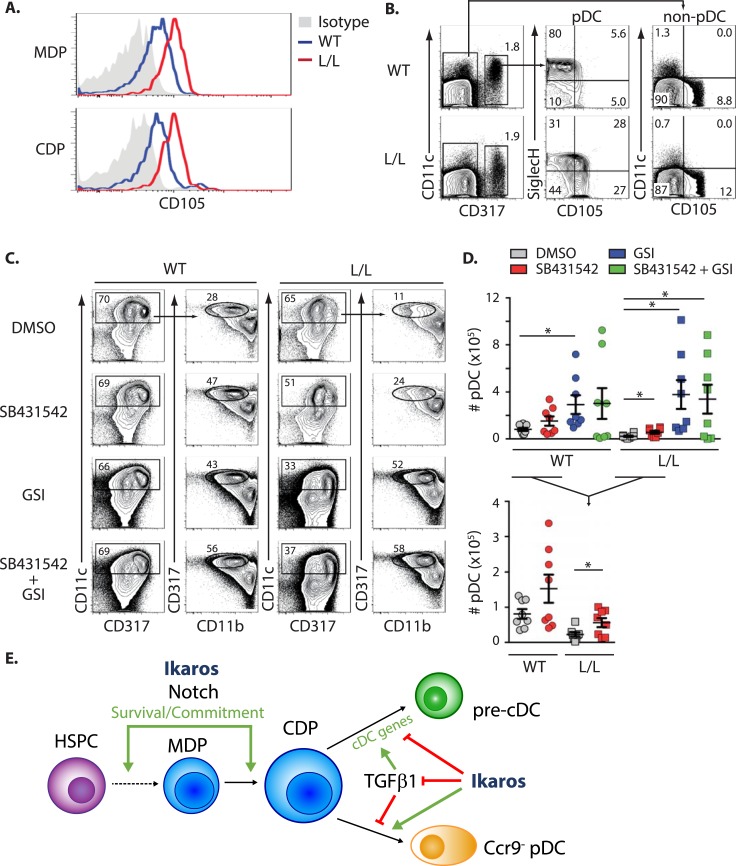
TGFβ1 activation inhibits pDC development from Ik^L/L^ CDPs. **(A)** CD105 expression on WT and Ik^L/L^ DC progenitors. **(B)** SiglecH vs. CD105 expression on BM pDCs (CD317^+^) and non-pDCs (CD317^-^). Representative of 4 independent experiments. **(C)** Effect of the TGFβR1 inhibitor SB431542 on pDC differentiation in Flt3L-supplemented WT and Ik^L/L^ BM cultures. Cells were treated at d0 with SB431542 and/or GSI and analyzed at d8. Percentages of cells in the corresponding gates are indicated. Representative of 4 independent experiments. **(D)** Number of pDCs obtained from experiments described in (C). Data of the SB431542 treatments are shown at a larger scale in the lower panel. Representative of 4 independent experiments; 2 mice per genotype per experiment; p values were obtained with a Student’s t-test. *p≤0.05. **(E)** Schematic representation of Ikaros function during DC development in the BM. Ikaros and Notch signaling are required for the onset of DC differentiation and the appearance of MDPs and CDPs. Later, in CDPs, Ikaros promotes pDC development by antagonizing TGFβ1 signaling and by repressing the cDC gene expression program. HSPC: Hematopoietic stem/progenitor cell.

To determine if the TGFβ pathway is activated in Ik^L/L^ CDPs, we studied the mRNA expression of genes encoding upstream components of this pathway. Although the level of transcripts encoding the type I and type II TGFβ receptors, and the downstream SMAD proteins, were similar between WT and Ik^L/L^ MDPs and CDPs, we found that the mRNA levels of *Eng*, encoding the type III TGFβ receptor Endoglin, was higher in Ik^L/L^ MDPs (2x) and CDPs (2.8x), regardless of GSI treatment (Fig 6A). Endoglin (CD105), is an auxiliary receptor for the TGFβ receptor complex, which has been shown to positively modulate TGFβ signaling [33]. Higher CD105 expression was also detected on Ik^L/L^ MDPs, CDPs and pDCs (Fig 7A and 7B). In contrast, CD105 levels were stable in other BM populations, including CD11c^+^CD317^-^ cDCs (Fig 7B), indicating that Endoglin expression is specifically increased in Ik^L/L^ pDCs and DC progenitors. In addition, we observed that Ikaros bound to the *Eng* locus in pre-B and DN3 cells, suggesting that it is an Ikaros target gene (S5A Fig).

Lastly, we analyzed the functional consequence of TGFβ inhibition on pDC development. WT and Ik^L/L^ BM cells were cultured with Flt3L for 8 days, in the presence or absence of a TGFβR1 inhibitor (SB431542), and/or GSI. SB431542 treatment alone did not affect total cell numbers (S5B Fig), but increased pDC numbers in both WT and Ik^L/L^ cultures (Fig 7C and 7D). In contrast, GSI treatment alone increased both total cell numbers and pDC numbers. The combination of SB431542 and GSI gave similar total and pDC numbers, compared with GSI alone. These results suggested that TGFβ inhibition promotes pDC differentiation in Ik^L/L^ CDPs.

## Discussion

Here we identify Ikaros as a promoter of early DC development. We show that Ikaros cooperates with Notch signaling to enhance the emergence and/or survival of MDPs and CDPs in the BM (Fig 7E). We also show that Ikaros is required to promote CDP differentiation and cell fate specification towards the pDC and cDC lineages, in large part by correctly regulating the expression of DC-specific target genes, and secondly, by antagonizing TGFβ function. These results indicate that the general absence of mature DCs in Ikaros null mice [13, 34], as well as the selective absence of pDCs in Ikaros hypomorphic animals [14], are due at least in part to CDP defects.

Our results suggest that Ikaros antagonizes a TGFβ-dependent gene expression program in CDPs. TGFβ was previously reported to skew CDP differentiation towards the cDC lineage at the expense of pDCs, in part because it induces the expression of Id2, which inhibits the master pDC regulator E2.2 [9, 35–37]. We show that Ikaros-deficient CDPs display a premature cDC gene expression signature, indicating that Ikaros represses the expression of mature cDC-associated genes in DC progenitors. In addition, Ikaros-deficient BM pDCs also display a cDC signature, suggesting that the mutant CDPs that commit to the pDC lineage continue to express a promiscuous cDC gene expression program. Neither *Id2* nor *E2*.*2* are affected at the mRNA level in Ikaros-deficient CDPs and pDCs, suggesting that Ikaros promotes CDP differentiation independently of E2.2.

How Ikaros antagonizes TGFβ function remains only partially understood. Certain TGFβ target genes are enriched among the genes deregulated by GSI in CDPs. Furthermore, Ikaros-deficient CDPs ectopically express high levels of endoglin which can potentiate TGFβ signaling [38]. Because no other TGFβ receptors or downstream SMAD factors are deregulated in these cells, endoglin upregulation probably plays an important role in activating the TGFβ pathway in the mutant DC progenitors. Interestingly, betaglycan, a type III TGFβ receptor closely related to endoglin in structure and function, is a substrate of γ-secretase, and GSI inhibits TGFβ2-mediated reporter gene expression via betaglycan inactivation in HepG2 cells [39]. γ-secretase cleavage of type III TGFβ receptors may therefore inhibit TGFβ receptor signaling in Ikaros-deficient cells. If true, this suggests that Ikaros may be a novel upstream regulator of TGFβ signaling.

In addition to its role in CDP differentiation, Ikaros is also required for MDP and CDP homeostasis. Observed only in compound mutants deficient for Ikaros and RBPJ where both populations are absent, our results demonstrate that Ikaros cooperates with Notch activation to maintain DC progenitor survival and/or expansion. Notch signaling by itself was previously found to promote DC development in vitro via up-regulation of the Frizzled family Wnt receptors [10], but the basis for its cooperation with Ikaros remains to be elucidated. We have reported that Ikaros antagonizes Notch function in T cells, and interacts directly with the activated Notch1 protein to control a set of common target genes [40]. Whether Ikaros and Notch regulate common genes in DC progenitors remains to be investigated. Other studies have suggested that the Notch and TGFβ pathways interact to regulate common genes. Indeed, *Hes1* is a common target of both pathways, because it is transcriptionally regulated by the Notch receptor intracellular domain or by Smad3 following TGFβ signaling [41]. In Ik^L/L^ cells, however, *Hes1* up-regulation was observed in pDCs but not in the more immature dendritic progenitors, suggesting that *Hes1* is differentially regulated by Notch and TGFβ activation in these populations.

Finally, our results with γ-secretase inhibitors are unexpected and intriguing, and indicate that these compounds can be exploited to enhance and rescue WT and Ikaros-deficient DC development in vitro, though the effects are stronger in the mutant cells. We showed that transient GSI treatment promotes the generation of CD11c^-^ cells, probably the upstream precursors of MDPs and CDPs, and pDC differentiation from CDPs. These actions suggest that GSI molecules might be considered as a potential treatment to enhance pDC function during certain viral infections, like chronic HIV or hepatitis C virus. Conversely, it will be important to test if GSI molecules might have a differentiating effect on BPDCN cancers, a rare and fatal leukemia with few options for treatment [42].

## Materials and methods

### Ethics statement

All mouse procedures were approved by the IGBMC Ethical Committee (Com'Eth); APAFIS#8752–20 170 1261 0337966 v2.

### Mice

The mouse lines used in this study were described previously: Ik^L/L^, Hes1-EmGFP^SAT^, RBPJ^f/f^ and R26-CreER(T2) [19, 23, 29, 30]. Mice were used between 6–9 weeks of age. To delete *Rbpj* in vivo, RBPJ^f/f^ R26-CreET(T2)^+^ or Ik^L/L^ RBPJ^f/f^ R26-CreET(T2)^+^ mice were injected intraperitoneally daily for 5 days with 75 mg/kg of tamoxifen dissolved in sunflower oil, and analyzed 10 days after the first injection.

### Cell culture

pDC cultures were performed as described [26]. Briefly, BM cells were seeded at 2x10^6^ cells/ml, and cultured in RPMI 1640 containing 10% fetal calf serum, 20 mM HEPES, 2 mM L-glutamine, 2 mM Sodium Pyruvate, 50 μM β-mercaptoethanol, 1x MEM non-essential amino acids, and antibiotics. Cultures were supplemented with conditioned medium from a Flt3L-producing cell line (B16-Flt3L) [43], or rFlt3L at 100 ng/ml (Peprotech). After 4d, half of the medium was replaced with fresh medium containing 2x Flt3L. GSI (Compound E, Calbiochem) and SB431542 (Selleckchem) were used at 5 μM. pDC cultures from DC progenitors were performed as above in 1 ml of Flt3L-supplemented medium using FACS-sorted Lin^-^Sca1^-^ckit^+^, MDPs, CDPs or CD115^-^CDPs from BM cells (CD45.2^+^) which were co-cultured with 10^6^ CD45.1^+^ whole BM cells. For CpG oligo-deoxynucleotide (ODN) stimulations, pDCs (CD11c^+^CD317^+^CD11b^-^) were sorted after 8 days of culture and stimulated at 2x10^6^ cells/ml in 96-well plates. CpG ODN 1585 or an ODN control (InvivoGen) were used at 2.5 μM. Cells were collected after 16h of stimulation.

### Transplantations

BM cells from donor mice (CD45.2^+^) were cultured with Flt3L in the presence or absence of GSI for 48h. 2x10^5^ cells from these cultures were then transplanted with 2x10^5^ supporting WT BM cells (CD45.1^+^) into lethally-irradiated (9 Gy) CD45.1^+^CD45.2^+^ recipient mice. Mice were sacrificed and analyzed 9 days after the transfer.

### RT-qPCR

RNA was extracted with the RNeasy (Qiagen) or Nucleospin RNA (Macherey-Nagel) kits, and reverse transcribed using Superscript II (Invitrogen). *Hes1*, *Ptcra* and *Hprt* were amplified using the QuantiTect SYBR green system with the Mm_Hes1_1SG, Mm_PtcrA_1SG and Mm_Hprt_1SG primer sets (Qiagen). *Ifna* mRNA was amplified using the SYBR green master mix (Roche) with 50 cycles of 10s 95°C, 30s 66°C, 15s 72°C. Primers used to amplify most of the *Ifna* subtypes were 5'-cctgctggctgtgaggaaata and 5'-gcacagggggctgtgtttct. Primers for *Ubiquitin* (*Ubb*) were 5'-tggctattaattattcggtctgcat and 5'-gcaagtggctagagtgcagagtaa. *Hes1* and *Ptcra* levels were normalized to that of *Hprt*, while *Ifna* expression was normalized to that of *Ubb*.

### Flow cytometry

We used the following antibodies: anti-CD11b (M1/70) eFluor450 or PE; anti-CD11c (N418) AlexaFluor700; anti-human/mouse CD45R (B220) eFluor650NC; anti- CD59 and Gr1 (RB6-8C5) biotin; anti-CD199 (CCR9) PE/Cy7; anti-CD317 (ebio927) AlexaFluor488 or eFluor450; anti-MHCII (M5/114.15.2) FITC or PE/Cy5; anti-Sca1 (D7) biotin (eBioscience); anti-CD3 (145-2C11) biotin; anti-CD4 (RM4-5) biotin; anti-CD8 (53–6.7) biotin; anti-CD11b (M1/70) biotin; anti-CD45.1 (A20) PE; anti-human/mouse CD45R (B220) biotin; anti-CD115 (c-fms) APC; anti-CD135 (A2F10) PE; anti-CD172a (P84) APC; anti-NK1.1 (PK136) biotin; anti-Ter119 biotin (BD Biosciences); anti-CD11c (N418) biotin or APC; anti-CD19 (6D5) biotin; anti-CD45.1 (A20) FITC; anti-CD45.2 (104.2) PE or AlexaFluor700; anti-CD105 (Endoglin) Alexa488; anti-CD117 (c-kit) APC/Cy7; anti-Ly49Q (2E6) PE (MBL); anti-SiglecH (551.3D3) PE (BioLegend); AlexaFluor™ 405 (InvitroGen) or AlexaFluor488 Streptavidin (Jackson ImmunoResearch). Lineage staining was performed using a mixture of anti-CD3, -CD4, -CD8, -CD19, -CD11b, -CD11c, -Gr1, -Ter119, -NK1.1 and -B220 antibodies for Lin, and anti-CD3, -CD19, -Ter119, -NK1.1 and -B220 for Lin*. Cells were analyzed on a LSRII analyzer (BD Biosciences) and sorted on a FACSAriaIISORP (BD Biosciences). Sort purity was >98%.

### Western blotting

Total protein extracts from 10^6^ BM cells were separated on SDS-PAGE gels. Immunoblots were analyzed with anti-RPBJ (T6719; Institute of Immunology, Japan), and anti-β-actin (A5441, Sigma) polyclonal antibodies. All secondary antibodies were horseradish conjugated (Santa Cruz, Jackson ImmunoResearch).

### Microarray analysis

Transcriptome analyses were performed with Affymetrix Gene ST 1.0 arrays. Unsupervised hierarchical clustering and K-means clustering were performed using Cluster 3. GSEA was performed using the GSEA 2.0 software [44, 45]. Microarray data are available in the GEO databank (GSE114108).

## Supporting information

S1 Fig GSI does not act on CD115^-^ CDPs to stimulate pDC differentiation. **(A)** Representative analysis of CD115^-^ CDPs from WT and Ik^L/L^ BM, by flow cytometry. **(B)** Relative numbers of CD115^-^ CDPs (as gated in A). ns: not significant (Student's t-test). **(C)** Experimental scheme: CD115^-^ CDPs from WT or Ik^L/L^ BM (CD45.2^+^) were cultured with supporting C57BL/6^CD45.1^ (CD45.1^+^) WT BM cells. Cultures were treated with GSI or DMSO at d0, and the percentage of CD45.2^+^ pDCs analyzed at d8. **(D)** Percentage of CD45.2^+^ pDCs (CD11c^+^CD317^+^CD11b^-^) after 8 days of co-culture. Data from cells of the same mouse treated with DMSO or GSI were connected by lines. Data from 3 independent experiments.
(EPS)

S2 Fig Frequency of GSI-treated pDCs after transplantation. Frequencies of pDCs (CD11c^+^CD317^+^CD11b^-^) from CD45.1^+^ BM and CD45.2^+^ GSI-treated WT and Ik^L/L^ cells in the BM of recipient mice 9 days post-transplantation.
(EPS)

S3 Fig Conditional deletion of RBPJ by tamoxifen in Ik^L/L^ mice. Western blot of RBPJ expression in total BM cells from Ikaros-RBPJ compound mutant mice. Actin was used as a loading control.
(EPS)

S4 Fig Gene expression changes in Ik^L/L^ CDPs. Transcriptome profiling of purified MDPs and CDPs from WT or Ik^L/L^ BM, treated beforehand with GSI or DMSO for 24h. **(A)** Hierarchical clustering of the genes from clusters I and III in Fig 6A, using Immgen transcriptome data for DC progenitors and mature subsets (GSE15907). **(B)** GSEA enrichment plots of genes up- or down-regulated in Ik^L/L^ CDPs compared with WT (clusters II and IV, and clusters I and III in Fig 6A, respectively). **(C)** GSEA enrichment plots of genes specifically up- or down-regulated in Ik^L/L^ pDCs compared with WT (FC>2; p≤0,05) [14]. In (B) and (C), the ranked gene list corresponds to the differential gene expression between WT cDCs and pDCs (Immgen GSE15907). NES: normalized enrichment score; FDR: false discovery rate. **(D)** Genome browser tracks showing Ikaros binding sites in the *Rel* and *Relb* loci in pre-B cells and DN3 thymocytes (GEO GSE114629 and GSE61148 accession numbers).
(EPS)

S5 Fig TGFβ1 signaling during pDC development in Ik^L/L^ CDPs. **(A)** Genome browser tracks showing Ikaros binding in the *Eng* locus in pre-B cells and DN3 thymocytes (GEO GSE114629 and GSE61148 accession numbers). **(B)** Total numbers of cells after 8 days of Flt3L-supplemented cultures of WT and Ik^L/L^ BM cells treated with SB431542 and/or GSI. See experiments shown in Fig 7C and 7D. Representative of 4 independent experiments; 2 mice per genotype per experiment; p values were obtained by a Student’s t-test. *p≤0.05; ***p≤0.001.
(EPS)

S1 Table FC of the 70 genes deregulated in Ik^L/L^ CDPs vs. WT cells, and sensitive to GSI treatment. (EPS)
